# Clinical Analysis of 152 Cases of Multiple Primary Malignant Tumors in 15,398 Patients with Malignant Tumors

**DOI:** 10.1371/journal.pone.0125754

**Published:** 2015-05-06

**Authors:** Zhihe Liu, Chunshui Liu, Wei Guo, Siyun Li, Ou Bai

**Affiliations:** 1 Department of Oncology, First Hospital of Jilin University, Changchun, China; 2 Department of Pediatrics, First Hospital of Jilin University, Changchun, China; University of Nebraska Medical Center, UNITED STATES

## Abstract

**Objectives:**

In this study, the etiology, clinical characteristics, and prognosis of multiple primary malignant tumors (MPMTs) were investigated. Furthermore, we analyzed the treatment factors associated with MPMTs.

**Methods:**

From 15,398 patients with malignant tumors presenting to The First Hospital of Jilin University, China, between January 2010 and December 2013, we identified and analyzed patients with MPMTs. Data were obtained retrospectively from the hospital database.

**Results:**

The prevalence of MPMTs in this study was 0.99% (152/15398): 51 cases were synchronous MPMTs, and 101 cases were metachronous MPMTs. The mean time between the first and second primary cancer was 43.1 months. In this population, MPMTs were observed more frequently in patients with head and neck tumors (5.65%) and urinary tumors (4.19%); the prevalence of MPMTs in these patients was over 4-fold greater than the prevalence of MPMTs in all patients (0.99%). There were no cases of MPMTs in 132 cases of nervous system tumors and 404 cases of multiple myeloma. Nearly 50% (45.4%) of patients with MPMTs did not receive chemotherapy or radiotherapy before the second primary cancer was diagnosed. Eighty-five patients with MPMTs were followed for more than 2 years, and the 2-year cumulative survival rate was 40.8%.

**Conclusions:**

In this study, the prevalence of MPMTs was 0.99% (152/15398), which is consistent with the Chinese literature. Patients with head and neck tumors or urinary tumors are at greater risk of developing MPMTs. In addition to radiotherapy or chemotherapy, this study suggests that other factors may contribute to MPMTs.

## Introduction

The life expectancy of patients with malignant tumors has increased in recent years as treatment options have improved. The 5-year survival rate is now nearly 66% [1] in all cancer patients.

With increased numbers of cured patients and long-term survivors, the risk of developing multiple primary malignant tumors is increasing. The term “multiple primary malignant tumors” was first used by Billroth in 1889, and the first paper describing MPMTs was published by Warren and Gates in 1932[2]. Since that time, the term “MPMTs” has been extensively used. MPMTs are defined as two or more independent primary malignancies of different histologies/origins in the same individual. MPMTs may be synchronous or metachronous. The term “synchronous” is used when the second primary cancer is diagnosed within 6 months of the primary cancer; “metachronous” is used when the second primary cancer is diagnosed more than 6 months after the diagnosis of the primary cancer. The incidence of MPMTs ranges from 0.4% to 21.0% [3–6] in various studies and different countries. Different factors have been suggested to explain the increased risk of a new malignant tumor in cancer patients, including exposure to chemotherapy and radiotherapy. In this study, we analyzed the etiology, clinical characteristics and prognosis of patients with MPMTs and examined the treatment factors associated with MPMTs.

## Materials and Methods

We retrospectively reviewed 15,398 patients with malignant tumors who presented to the First Hospital of Jilin University, Changchun City, Jilin Province, China between January 2010 and December 2013. Among the 15,398 patients with malignant tumors, we identified 152 cases of MPMTs. All patients showed histopathological evidence of first and second primary cancers. Cases in which the second primary cancer developed in the same organ or system were included in this study, but, sarcomas and soft tissue tumors were not included in the study.

All subjects signed an informed consent form prior to the initiation of the study, which was approved by the Ethics Committee of the First Hospital of Jilin University.

The 15,398 patients with malignant tumors exhibited digestive system tumors, lung cancer, breast cancer, leukemia/lymphoma, gynecological tumors, multiple myeloma, head and neck tumors, urinary tumors and nervous system tumors. The digestive system tumors included gastric cancer, liver cancer, esophageal cancer, pancreatic cancer and colorectal cancer. Gynecological tumors included cervical cancer, ovarian cancer and choriocarcinoma. Urinary tumors included bladder cancer, renal carcinoma, prostate cancer and testicular cancer. Head and neck tumors included nasopharyngeal carcinoma, laryngeal carcinoma, thyroid cancer and carcinoma of the parotid gland.

For the diagnosis of multiple primary malignant tumors, we used the criteria proposed by Warren and Gates [2]. The 152 cases of MPMTs complied with these criteria. In terms of “synchronous” with no interval time during two tumors; the tumor, being associated with the cause for the visits of patients and firstly diagnosed with cancer, was defined as the first primary cancer; nevertheless, the other tumor was defined as the second primary cancer. At last, patients with survival <6 months were defined as patients with single cancer if we didn’t find the second primary cancer before they died.

Overall survival (OS) was measured from diagnosis to the date of last follow-up or death from any cause. SPSS 16.0 was used to analyze OS, and a two-sided P value ≤0.05 was considered to be significant.

Treatment factors included surgery, chemotherapy, radiotherapy, post-chemotherapy, post-radiotherapy, post-chemoradiotherapy, and chemoradiotherapy.

## Results

### Characteristics of the participants

Among the 15,398 patients with malignant tumors, 152 patients had MPMTs. Of these 152 patients, 69 were males and 83 were females; thus, the female to male ratio was nearly 1.2. The median age of the 152 MPMT patients at the time of diagnosis of the first primary cancer was 59 years in the total population (range 32–83), 61 years in men, and 59 years in women. More than 50% of the MPMT patients were 50 to 70 years old (50.6%).

### Prevalence

**Fig 1:**
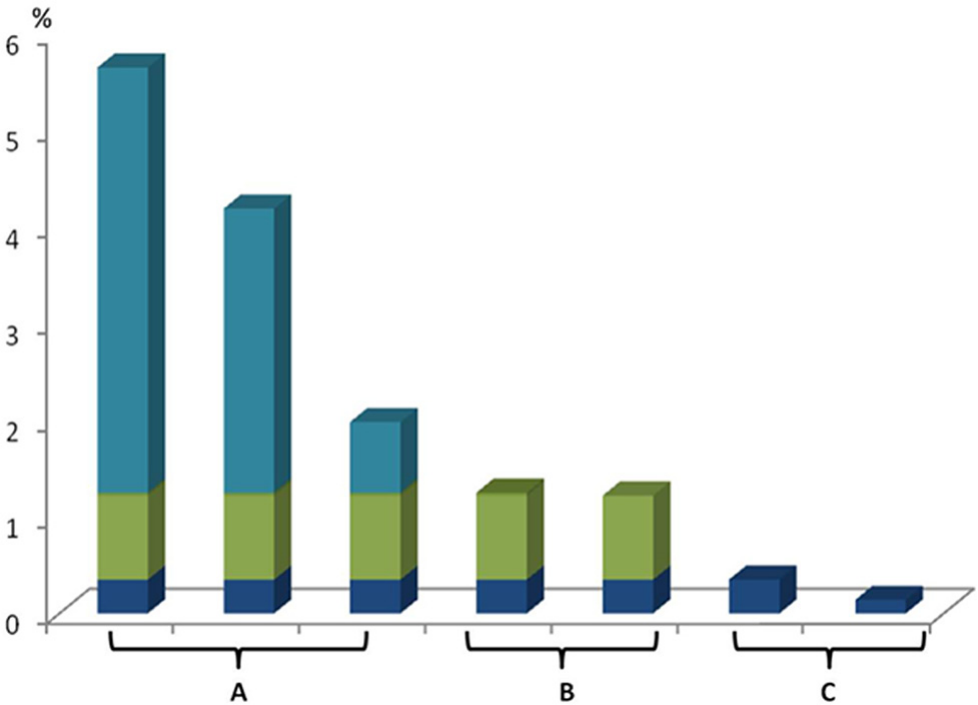
The ratios of MPMTs in different systems. 0.99% refers to the prevalence of MPMTs in 15,398 patients with malignant tumors. Group A included patients with head and neck cancer (5.65% versus 0.99%; P = 0.000), urinary tumors (4.19% versus 0.99%; P = 0.000) and gynecological tumors (1.98% versus 0.99%; P = 0.008). Group B included patients with breast cancer (1.22% versus 0.99%; P = 0.241) and digestive system tumors (1.25% versus 0.99%; P = 0.151). Group C included patients with lung cancer (0.35% versus 0.99%; P = 0.000) and leukemia/lymphoma (0.14% versus 0.99%; P = 0.000).

Out of 15,398 patients with malignant tumors, 152 patients had MPMTs, corresponding to an overall prevalence of 0.99% (152/15,398). The 15,398 patients with malignant tumors include patients with tumors in different organs, including digestive system tumors and lung cancer, as mentioned previously Table 1. The patients were classified into three groups based on the prevalence of MPMTs (Fig 1): group A included patients with head and neck cancer, urinary tumors and gynecological tumors; the prevalence of MPMTs in these patients was greater than 2-fold of that in the total population (0.99%; P<0.05). Group B included patients with digestive system tumors and breast cancer, and the prevalence of MPMTs in these patients was only slightly higher than that in the total patient population (P>0.05). Group C included lung cancer and leukemia/lymphoma patients, and the prevalence of MPMTs in these patients was significantly lower than that in the total patient population (P<0.05). There were no cases of MPMTs in the 404 cases of multiple myeloma or the 158 cases of nervous system tumors.

**Table 1:** Cases and ratios of MPMTs in different systems.

Malignant tumor	N	MPMTs(N)	Ratio (%)
**Lung cancer**	**3389**	**12**	**0.35**
**Breast cancer**	**3028**	**37**	**1.22**
**Leukemia/lymphoma**	**2960**	**4**	**0.14**
**Gynecological tumors**	**757**	**15**	**1.98**
**Digestive system tumors**	**4014**	**50**	**1.25**
**Urinary tumors**	**334**	**14**	**4.19**
**Head and neck cancer**	**354**	**20**	**5.65**
**Nervous system tumors**	**158**	**0**	**0.00**
**Multiple myeloma**	**404**	**0**	**0.00**
**Total**	**15398**	**152**	**0.99**

In 152 MPMTs, the ratio of top 3 in second primary cancer was digestive system tumors (36.8%, 56/152), lung cancer (18.4%, 28/152) and leukemia/lymphoma (13.2%, 20/152), respectively; and the ratio of other second primary cancers in 152 MPMTs show below (Table 2).

**Table 2:** The distribution of second primary cancer in 152 MPMTs.

Malignant tumor	Second primary cancer N(ratio)
**Digestive system tumor**	**56(36.8%)**
**Breast cancer**	**12(7.9%)**
**Head-neck cancer**	**13(8.6%)**
**Urinary system tumor**	**8(5.3%)**
**Gynecological tumors**	**15(9.9%)**
**Lung cancer**	**28(18.4%)**
**Leukemia/lymphoma**	**20(13.2%)**

### Synchronous and metachronous MPMTs

The 152 cases of MPMTs included 51 cases of synchronous MPMTs and 101 cases of metachronous MPMTs. The mean interval between the first and second primary cancer was 43.1 months (range, 0–349 months).

### Treatment factors

Of the 51 patients with synchronous MPMTs, 82.4% (42/51) were diagnosed when they were first admitted to the hospital. Of the 101 patients with metachronous MPMTs, 26.7% (27/101) had been treated with surgery alone before the second primary cancer was diagnosed, and 73.3% (74/101) underwent chemotherapy or radiotherapy before the MPMTs were diagnosed Table 3. In general, 45.4% (69/152) of MPMT patients received no chemotherapy or radiotherapy.

**Table 3:** Treatment histories of 101 cases of metachronous MPMTs.

Treatment	First primary cancer N(ratio)
**Surgery**	**27(26.7%)**
**Chemotherapy**	**57(56.4%)**
**Radiotherapy**	**7(7.0%)**
**Chemo-radiotherapy**	**10(9.9%)**

### Prognosis

**Fig 2:**
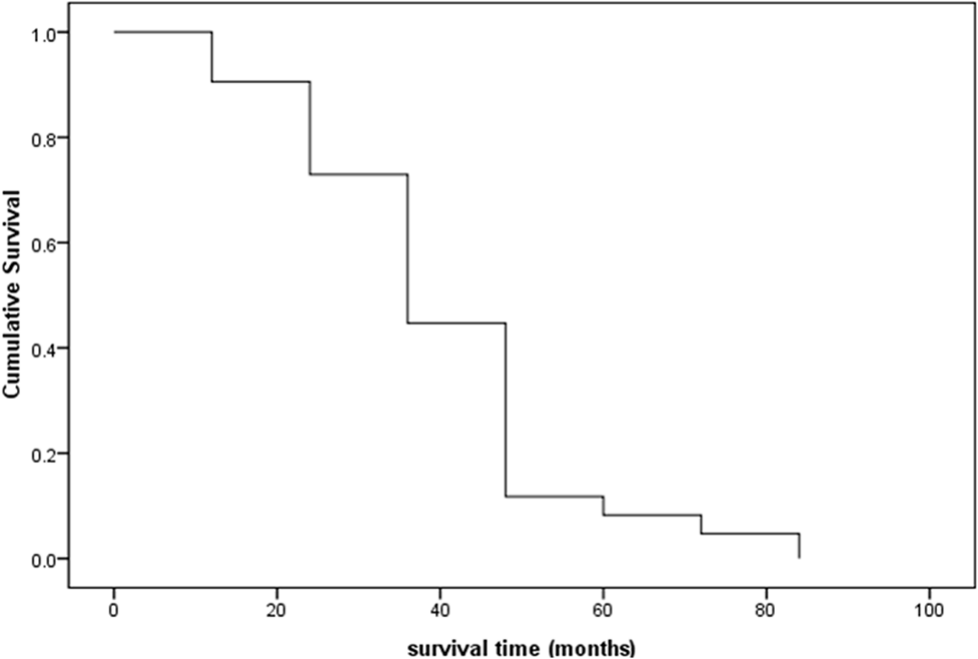
The survival curve of 85 MPMTs.

Up to December 2013, 152 cases of MPMTs had been followed up effectively, and 24 patients had died. A total of 85 patients with MPMTs were followed up for more than 2 years, and the 2-year cumulative survival rate was 40.8% (Fig 2).

## Discussion

In this study, females were slightly more likely to suffer from MPMTs than males. The female to male was 1.2, which is similar to what was reported in China [3,7–8], but different from what was reported in Japan. It is because that the majority of patients exhibited primary tumors of the digestive system in Japan, the majority of Chinese female patients suffered from breast cancer or gynecologic malignant tumors.

There were 354 cases of head and neck cancer in this study, and the prevalence of MPMTs among these cases was 5.6% (20/354). In 20 cases of MPMTs, the first primary cancers were laryngeal carcinoma (7 cases) and thyroid cancer (9 cases). Nine cases of thyroid cancer and 3 cases of laryngeal cancer did not receive chemotherapy or radiotherapy before the second primary cancer was diagnosed. We believe that in addition to chemotherapy and radiotherapy, there other causes of MPMTs in patients with head and neck cancer may exist, but the precise mechanism remains to be determined. We further investigated the survival of the head-neck tumors, 15 of 20 cases of head-neck tumors, at the end of the follow-up, survived, whose mean survival time followed up more than two years was 26.3 months, which was significantly higher than those of patients with lung cancer, given the results above, we believed that the survival time of patients with head-neck tumors may be a potential factor for suffering from MPMTs. Therefore, doctors should pay greater attention to the development of MPMTs in patients with head and neck cancer.

Of 15398 patients, there were 3389 lung cancer patients and 2960 leukemia/lymphoma patients, but, these patients rarely experienced a second malignant diagnosis in this study; however, of second primary cancer in 152 MPMTs, the ratio of lung cancer and leukemia/lymphoma was 18.4% and 13.2%, respectively, they accounted for a substantial component of the second malignancy in this population. We consider that the phenomenon may be associated with a short time span from diagnosis to death and the characteristics of tumors. As we all know, lung cancer is highly aggressive tumor, median overall survival of those patients treated with chemotherapy is dismal, which is almost less than 2 years. Because of a short time span from diagnosis to death of the first primary cancer patients, commonly the case for lung cancer, then a second diagnosis of cancer is less likely because of limited time available for a second cancer to become apparent. Thus lung cancer is reasonably often associated with other malignancies but rarely when they are the patients as first malignancy, this concept that most likely also apply to other cancers that are very aggressive, such as leukemia/lymphoma.

MPMTs are a rare phenomenon in tumorigenesis. In recent years, several papers examining MPMTs have been published, but the reported prevalence differs significantly. The prevalence of MPMTs in foreign studies ranged from 11.0% to 21.0% [4–5]; however, the prevalence of MPMTs was reported to be 0.4%-2.4% [9] in China. In our dataset obtained from the First Hospital of Jilin University, China between January 2010 and November 2013, the prevalence of MPMTs was 0.99%, which is similar to the prevalence in China but less than that reported in other countries, such as Sweden [5]. The reasons for the lower prevalence in China may include the following: 1. Misdiagnosis: Doctors are not on the lookout for MPMTs, and some patients who suffer from MPMTs may be misdiagnosed with metastatic carcinoma. 2. Difficulty of detection: The primary cancer may be very small and difficult to detect at the time of presentation. Furthermore, autopsies are rarely performed, which can also result in misdiagnosis. 3. Misregistration: Some patients may develop MPMTs during the progression of their primary cancer but fail to present to the hospital in a timely manner, leading to the lack of documentation of their MPMTs. 4. Time span: Time span of the study, starting recently in 2010, covers only 3 years, which maybe explain the difference in prevalence when compared to the reported prevalence of multiple malignancies, reference 7, because some second primary cancer may not easily detected during this time span. 5. Population: The study is very limited in population covering partly Jilin province, not China, what’s more, the bottom line data of foreign studies mentioned above is from population based cancer registry in country or many cancer centers with a long history of highly reliable data on cancer diagnosis, we concede that this difference may also affect the prevalence of MPMTs in the study. 6. Sarcomas and soft tissue tumors: Our oncology center didn’t receive patients with sarcoma or soft tissue tumor before 2013, so sarcomas and soft tissue tumors were not included in this study, but, sarcomas and soft tissue tumors were included in published literatures, such as reference 7, we think this disadvantage may also affect the prevalence of MPMTs in this study.

The etiology of MPMTs remains unclear. Curtis RE al. [10] studied leukemia following chemotherapy for breast cancer among patients diagnosed during 1973–1985 within the population-based tumor registries in the Surveillance, Epidemiology, and End Results, Program. They found that among 20 cases (17 incident leukemia and 3 deaths due to preleukemia) and 60 matched controls, alkylating agents were linked to an 11.9-fold risk of ANLL (acute nonlymphocytic leukemia) and preleukemia (95% confidence interval = 2.6–55). Chemotherapy regiments including melphalan were related to a higher risk of leukemic conditions than those including cyclophosphamide. Haas J.F et al [11] conducted a case-control study to determine whether the development of leukemia was associated with chemotherapy for neoplasms of the ovary or breast, in a population where most such chemotherapy consisted of cyclophosphamide alone. The study indicated that the relative risk for acute leukemia following treatment with cyclophosphamide alone was significantly elevated (P<0.05), at 14.6 for ovarian tumor patients and 2.7 for breast cancer patients. Harvey and Brinton [12] examined the data for 41,109 women diagnosed with breast cancer between 1935 and 1982 and found that women treated with radiation were at a higher risk of developing a second breast cancer than were nonirradiated women (SIR, 3.9versus2.8). Murakamiet al. [13] observed an excess risk of contralateral breast cancer in the radiotherapy group only among those diagnosed at 10 or more years after the first breast cancer diagnosis (SIR, 7.6) compared with the nonradiotherapy group (SIR, 2.9), whereas the overall SIR was 3.8 for the radiotherapy group and 4.8 for the nonradiotherapy group. Alfred I al. [14] investigated whether there was a similar relationship between breast cancer, colorectal cancer, and prostate cancer in men. In this study, that breast and colorectal cancer appear to co-occur in women but not in men suggests that certain risk factors may be operating in women that are not present in men. The most obvious explanation of these findings suggests that reproductive hormonal factors play a significant role in the cause of these cancers in women. Heidi D al. [15] assessed the benefits and harms of hormone replacement therapy for the primary prevention of cancer, they find that harms include breast cancer with 5 or more years of use. Wu A.H al. [16] studied the etiology of colorectal cancer in a cohort of 11,888 residents of a retirement community. After four and one-half years of follow up, 58 male and 68 female incident colorectal cancers were identified, what’s more, daily alcohol drinkers experienced nearly a two-fold increase in risk (2 sided P = 0.002). Samuel J al. [17] determined the impact of index primary tumor site on second primary malignancies (SPM) risk and explored factors potentially affecting this risk within a large prospectively accrued cohort of patients with index squamous cell carcinoma of the head and neck, they found that former-smokers had a 50% greater risk of SPM and current-smokers had a 100% greater risk of SPM than never-smokers (P trend = .008). Schatzkin A al. [18] investigated the extent to which the diet and cancer hypothesis was supported by data from the Surveillance, Epidemiology, and End Results (SEER) Program on multiple primary associations, the results of the study were that Of the eight multiple primary associations among diet-related cancers that were possible in men and women, relative risks (RR) of a second diet-related primary cancer developing after a first diet-related primary ranged from 1.06 to 1.43. Morita M al. [19] conducted the study to elucidate the relationship between the number of carcinomas and risk factors, and the study demonstrated that 27% of patients with three or more cancers had a close relative with upper aerodigestive tract (UADT) or lung cancer, while the incidence was 7% in the control, regarding family history. The present study indicated that the etiology and pathogenesis of MPMTs may involve other factors in addition to factors above mentioned.

In 15398 patients, there were 404 patients with multiple myeloma and 158 patients with nervous system tumor, but, none of them experienced a second malignant diagnosis, what’s more, except chemotherapy, these patients were not involved with factors leading to MPMTs mentioned above, as a result, the precise etiology and pathogenesis of MPMTs warrant further study.

All 152 patients with MPMTs were followed-up through November 2013. The 2- and 5-year survival rates were 40.8% and 4.6%, respectively, highlighting the need for doctors to be aware of MPMTs.

## Conclusions

Out of 15,398 cases of malignant tumors, we found 152 cases of MPMTs. Thus, the prevalence of MPMTs at our center was 0.99% (152/15398), which is consistent with that reported in the Chinese literature. Doctors should pay particular attention to the possibility of MPMT occurrence in patients with head and neck tumors or urinary tumors. In addition to radiotherapy and chemotherapy, several other factors likely contribute to MPMTs.
